# How Plastic Can Phenotypic Plasticity Be? The Branching Coral *Stylophora pistillata* as a Model System

**DOI:** 10.1371/journal.pone.0000644

**Published:** 2007-07-25

**Authors:** Lee Shaish, Avigdor Abelson, Baruch Rinkevich

**Affiliations:** 1 Israel Oceanographic and Limnological Research, National Institute of Oceanography, Haifa, Israel; 2 Zoology Department, Tel-Aviv University, Ramat Aviv, Israel; Wellcome Trust Sanger Institute, United Kingdom

## Abstract

Phenotypic plasticity enables multicellular organisms to adjust morphologies and various life history traits to variable environmental challenges. Here, we elucidate fixed and plastic architectural rules for colony astogeny in multiple types of colonial ramets, propagated by cutting from genets of the branching coral *Stylophora pistillata* from Eilat, the Red Sea. We examined 16 morphometric parameters on 136 one-year old *S. pistillata* colonies (of seven genotypes), originating from small fragments belonging, each, to one of three single-branch types (single tips, start-up, and advanced bifurcating tips) or to structural preparative manipulations (representing a single or two growth axes). Experiments were guided by the rationale that in colonial forms, complexity of evolving phenotypic plasticity can be associated with a degree of structural modularity, where shapes are approached by erecting iterative growth patterns at different levels of coral-colony organization. Analyses revealed plastic morphometric characters at branch level, and predetermined morphometric traits at colony level (only single trait exhibited plasticity under extreme manipulation state). Therefore, under the experimental manipulations of this study, phenotypic plasticity in *S. pistillata* appears to be related to branch level of organization, whereas colony traits are controlled by predetermined genetic architectural rules. Each level of organization undergoes its own mode of astogeny. However, depending on the original ramet structure, the spherical 3-D colonial architecture in this species is orchestrated and assembled by both developmental trajectories at the branch level, and traits at the colony level of organization. In nature, branching colonial forms are often subjected to harsh environmental conditions that cause fragmentation of colony into ramets of different sizes and structures. Developmental traits that are plastic, responding to fragment structure and are not predetermine in controlling astogeny, allow formation of species-specific architecture product through integrated but variable developmental routes. This adaptive plasticity or regeneration is an efficient mechanism by which isolated fragments of branching coral species cope with external environmental forces.

## Introduction

Phenotypic plasticity, the multiple-phenotype expressions by a single genotype in response to environmental forces [1], [2], enables multicellular organisms of all taxa to adjust morphologies, behaviors, physiologies and various life history traits to variable environmental encounters. Rich literature has confirmed, above any dispute, expressions of morphological plasticity through genotype-by-environmental interactions within the lifespan of a single organism. Such plastic variations in environmentally dependent morphological characters had also been documented in reef corals [3], [4], [5], [6], [7], [8], [9], [10], [11], [12], [13], [14], [15]

Coral colonies are made of multiple genetically identical modules (polyps) that are physiologically integrated [16]. Like other sessile colonial organisms [17], corals may generate extremely broad plasticity of phenotypic traits, changing their morphologies through accretion of new primary modules (polyps; the polyp level of organization) placed above existing phenotypic stable structures. In branching forms, two higher levels of organization exist. At some yet unidentified stage, a second type module, the branch, emerges, a phenomenon based on coordination between the primary modules. Despite the relative morphological simplicity of each module (at both, the polyp- and the branch-module levels), branching corals may generate complex architectures, at a third level of organization (the colony [18], [19]), that are either conserved or changed during the lifespan of any specific colony. Whereas some studies contend that coral plastic formation is driven entirely, or mainly, by environmental factors [12], [20], [21], [22], [23], [24], other studies elucidate the importance of genetically predetermined traits in construction of colonial architecture [8], [18], [19], [25], [26], [27]. Branching coral species, while altering between plastic morphological traits, present characteristic species-specific architectural rules and branching patterns that are expressed in harmony between modules and levels of organization; all for the assemblage of final colonial landscape. A fundamental, yet unsolved, question is type and leverage of freedom such a system has, in search for an optimal architecture under altering sets of environmental and biological challenges (considering claims that morphological plasticity in corals is either strictly adoptive and/or stems from absence of genetic canalization in morphological traits [28], [29]) .

Astogeny of the branching species *Stylophora pistillata* from the Red Sea is thought to be genetically controlled and determinant [18], [30], [31]. Colony architecture in this species reflects a single common astogenic plan characterized by a continuum of architectural designs with several distinct stages, each marked by specific morphometric parameters and traits [31]. Using 3-D accretive growth patterns, astogeny in *S. pistillata* colonies appears as a basic axial bifurcating rod-like growth, supplemented by lateral growing branches (sub-apical branches, sensu [19]), altogether featuring a suite of architectural rules on three levels of organization, the polyp, the branch and the colony levels. Integration of up-growing bifurcating branches emerges through dichotomous splitting at the branch tip (formation of two sister branches) and growth trajectories along inward- and outward-facing lateral branches [18], [30], [32]. Above observations, reveal evolutionary fixed robustness of colonial architecture that gives rise to the sphere-like typical colonial structure of this species [18], [30].

Morphological traits in *S. pistillata* colonies represent phenotypic plasticity tightly corresponding to environmental gradients [24], [31] that shape dynamically colonial architecture. This important property of colony design in *S. pistillata* could be expressed by morphometric parameters related to a single, or several levels, of colonial organization (the polyp, the branch, the colony architectural levels). This species, therefore, can be used as a model system for evaluating genetics vs. morphologic plasticity in forming colonial landscapes of stony corals, including ultimate and proximate causes of morphogenesis (sensu [33]). Clearly, morphological plasticity is advantageous for it allows a genotype to respond in a broader fashion to harsh environmental conditions. However, in branching forms the fitness of unalike fragment type architectures towards a complete regeneration of the colony structure has not been yet evaluated.

The present study focuses on fixed and plastic architectural rules for colony astogeny in colonies of *Stylophora pistillata* from Eilat. We analyzed morphometric parameters on three types of branches (crowned by single tips, start-up and advanced bifurcating tips) and manipulated artificial geometric settings (with a single or two growth axes). Results revealed that under the experimental manipulations of this study, and within an astogeny window of one year, phenotypic plasticity in *S. pistillata* appears to be restricted to the branch level of organization. Unless under extreme manipulation forces, the colony level of organization is controlled by non-plastic architectural rules (the species-specific blueprint rules; sensu ref. 18). Each level of organization, therefore, undergoes its own mode of astogeny.

## Materials and Methods

**Figure 1:**
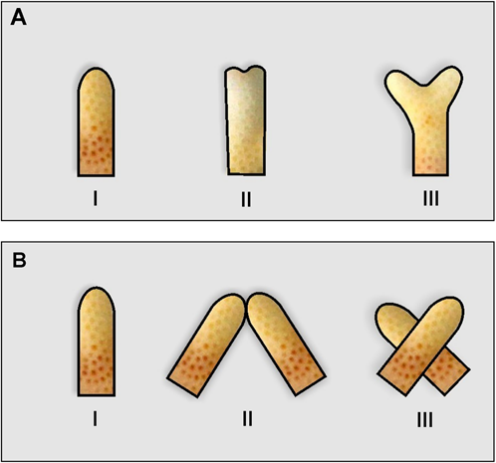
Schematic illustrations for initial shapes of *S. pistillata* branches (A) Experiment 1: setting I, single-tip branches; setting II, initial dichotomous branches; setting III, dichotomous tip branches. (B) Experiment 2: setting I, single-tip branches; preparative II, pairs of isogeneic branches with tip-to-tip contacts creating structures with two bases each (fused double tips); preparative III, pairs of isogeneic branches with center-to-center contacts, creating shapes with two tips and two bases each (crossed double tips).

Experiments were conducted in front of the H. Steinitz Marine Biology Laboratory in Eilat, the Northern Red Sea. Seven large *S. pistillata* colonies growing apart (>10 m) from each other at depth of 7 m (15–20 cm diameter each; marked by letters A to G) were carefully detached from substrates by chisel and hammer. Each colony represented only a single genet [18], [20], as colonial fragments of this species in Eilat do not brace development (Rinkevich, unpubl.). Following collection, colonies were incubated *in situ* in alizarin Red S solution (15 ppm; 12 h; following [30]) in transparent plastic bags. Branch tips, the major site of calcification, were labeled by red color. New deposits of calcium carbonate appeared as white zones above the red lines. Branches (2–4 cm long) were removed from all colonies by wire cutter following two weeks of post-labeling acclimation period. The seven *S. pistillata* genets were used in two sets of experiments. In the first (Fig. 1a), 30 branches were removed from each colony of genets A–D; 10 single-tip branches (group I), 10 initial dichotomous branches (each dichotomous initiative was less than 2 mm long; group II) and 10 well developed dichotomous tip branches (more than 2 mm long; group III), in total 120 branches. In the second set of experiments (Fig. 1b), 40 single-tip branches (of same size) were removed from each genotype E–G. Ramets from each of these three genotypes were distributed, haphazardly, between three treatment (preparative) groups: (I) control, single tip branches; (II) pairs of isogeneic branches arranged in tip-to-tip contacts, creating arrowhead-like structures with two bases and a single tip (fused double tips; fusions developed within less than 1 month of intimate contacts); (III) isogeneic pairs of branches with branch center-to-center contacts, creating X-like shapes with two tips and two bases each (crossed double tips). Any particular genet provided eight replicates for each preparative (n = 72 preparative for the three colonies). All ramets were attached by plastic clips to underwater nursery tables, placed at 7 m depth, under identical *in situ* conditions. After one year of growth in the field, 88 branches survived the first experimental set and 48 preparative survived the second experiment. The colonies were then brought to the laboratory and their tissues removed by immersion in household bleach for 24 h [34].

Sixteen morphometric parameters of each of the 136 colonies (MPs; [31]; Table 1) were measured and analyzed. These MPs differed in their state of complexity and level of integration, exhibiting various categories of phenotypic modularity (sensu [19]). Ten MPs described the colonial traits (final colony height, added height, percentage of height added, total number of branches, ecological volume, the ratio of skeletal volume to ecological volume, branch spacing, colony width or the lateral dimension of the colony, weight added and Ω, the order of colony complexity; see Table 1 for detailed definition). Six MPs described the branch traits (branch average length, up-growing and lateral-growing branches, branch order, total bifurcations and tip-born branches; Table 1). Branch order follows the Reverse Strahler order system [35], by which branches are ranked based on the hierarchical number of branching events starting from the original, primary branch [36]. In an isolated fragment, the stem is ranked 1 (in preparative of fused branches tip and crossed branches, both branches forming the initial shape are given the order 1); each secondary branch is ranked 2; branches that grow from the secondary branches are ranked 3, and so on.

**Table 1:** Morphometric parameters (MPs) considered as representing architectural rules important in *S. pistillata* colony astogeny

Morphometric character		Description	Level of organization	Way of measuring/calculating
**1**	**H**	Final colony height (mm)	Colony/genet	Measured from the substrate to the highest ramets' point [55].
**2**	**ΔH**	Height added (mm)	Colony/genet	(H-Ho); Vertical growth added after one year. Initial height (H0) was measured from the substrate to the alizarin mark [30].
**3**	**ΔH%**	Height added (%)	Colony/genet	Calculated as: (H-H0)/H0.
**4**	**nB**	Total no. of branches	Colony/genet	Total number of branches, including the initial branch [21], [12].
**5**	**Ev**	Ecological volume (mm^3^)	Colony/genet	Sum volumes of skeletons and spaces between the branches. πHr^2^; r = width+length / 4. Width and length, following Shais *et al*. [31],
**6**	**Sv/Ev**	Skeletal to ecological volumes ratio	Colony/genet	Sum of all branch volumes (each branch is calculated as a cylinder) divided by the ecological volume.
**7**	**Ev/nB**	Branch spacing (mm^3^)	Colony/genet	Ev/nB represents ecological volume per branch.
**8**	**Le**	Lateral dimension (mm)	Colony/genet	Colony width, maxial axis between two opposing LGBs
**9**	**ΔW**	Weight added (gr)	Colony/genet	W-W0; W0 = Initial weight, obtained after removal of all branches developed above the alizarin line [30], W = Weight after 1 year.
**10**	**Ω**	The order of colony complexity	Colony/genet	Numbers represent the highest reached hierarchical branch order. “Reverse Strahler Order” [35]; numbers represent the highest hierarchical branch order that a specific colony reached.
**11**	**BaL**	Branch average length (mm)	Branch/ramet	Lengths were measured by digital caliper to the nearest 0.01 mm. Average values were obtained following [21].
**12**	**%Nx = N1-N4**	Branches order (%)	Branch/ramet	Number of branches of each hierarchical order as part of the total number of branches (Reverse Strahler Order method; [35].
**13**	**%DI**	Dichotomous branches (%)	Branch/ramet	The number of branch-bifurcations divided by nB
**14**	**%SI**	Lateral branches (%)	Branch/ramet	The number of lateral branches divided by nB
**15**	**%UGB**	Up growing branches (%)	Branch/ramet	UGBs divided by nB
**16**	**%TBB**	Tip-born branches (%)	Branch/ramet	Branches originated from branch tips out of total number of new branches

Data analysis was performed by multivariate analysis methods using PRIMER5 software [37], [38]. Data was standardized by rescaling each category, which assured that each of the characters contributed equally to the overall information on the colony's morphological structure. A Bray-Curtis similarity matrix was used to compare the similarities between the genotypes in each experiment separately [31].

At first, Draftsman plot analysis was performed on the 16 MPs [31] in order to exclude parameters sharing high degree of correlation with each other (p>0.95), so that they would not add information to the data analysis [38]. Then, 2-D non-metric MDS (Multi Dimensional Scaling) algorithm was used on the Bray-Curtis similarity matrix to see whether the new colonies were grouped together according to the genet factor or according to the fragment shape (experimental group). In this analysis, points on a 2-D graph represented the genets or the experimental groups. The closer the points are the higher is their similarity [38].

ANOSIM (analysis of similarity test) was used to test the null hypothesis that primary shape of each developed colony, as represented by its morphometric characters, was similar to other colonies' shape in the same experimental group. The similarity between colonial replicates (ramets) of the same genet or of different experimental groups was represented by R values. When R>0.5 the genets, but not the ramets within a genet, differed from each other; when R<0.5, both the ramets and the genets did not differ from each other [37].

Univariate analysis was preformed, separately, on each morphometric parameter, using ANOVA (analysis of variance), with STATISTICA software. In these tests, the preliminary assumption was the existence of homogeneity of variance and not normal distribution [39]. Homogeneity of variance was checked using Levene's test. In cases were no homogeneity of variance was found, transformation of square root or Log 10 was preformed on the data [40]. When transformation did not reveal homogeneity of variance, we used the aparametric test Kruskal-Wallis.

## Results

**Figure 2:**
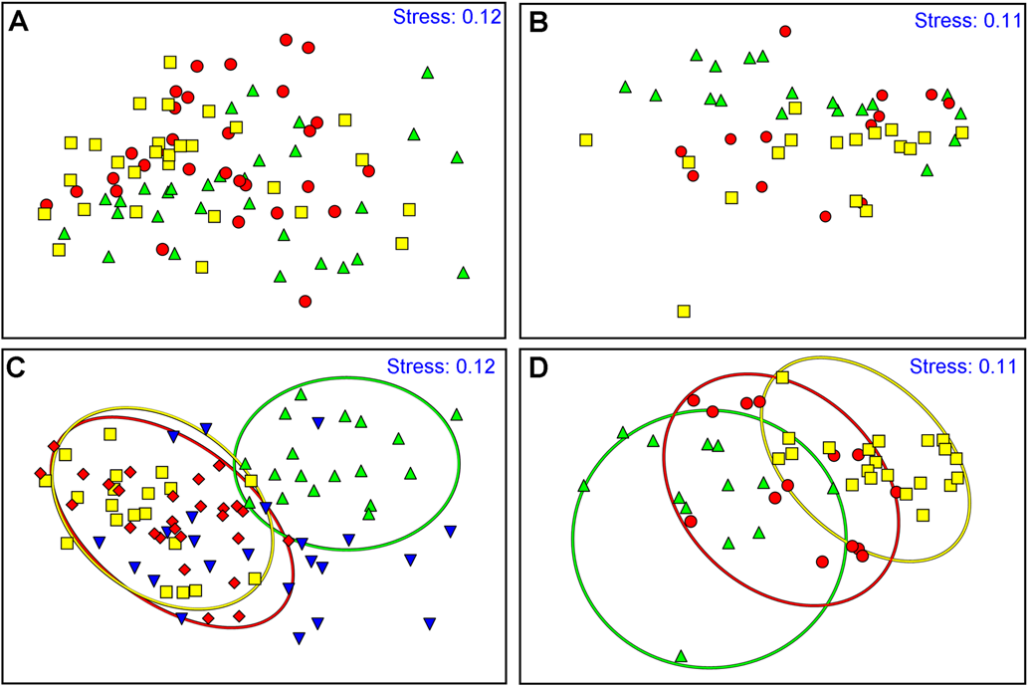
Two-dimensional MDS ordination for all (n = 136) daughter colonies. A and B are factored by the different settings; C and D are factored by genotypes (A) Experiment 1, triangle = setting I, single tip; circle = setting II, initial dichotomous tip; square = setting III, dichotomous tip. (B) Experiment 2, triangle = l setting I, single tip; circle = setting II, fused double tip; square = setting III, crossed double tip. (C) Experiment one, triangle = genotype A, tip-down triangle, donor colony B, square = genotype C, diamond = genotype D. (D) Experiment two, triangle = genotype E; circle = genotype F; square = genotype G.

**Figure 3:**
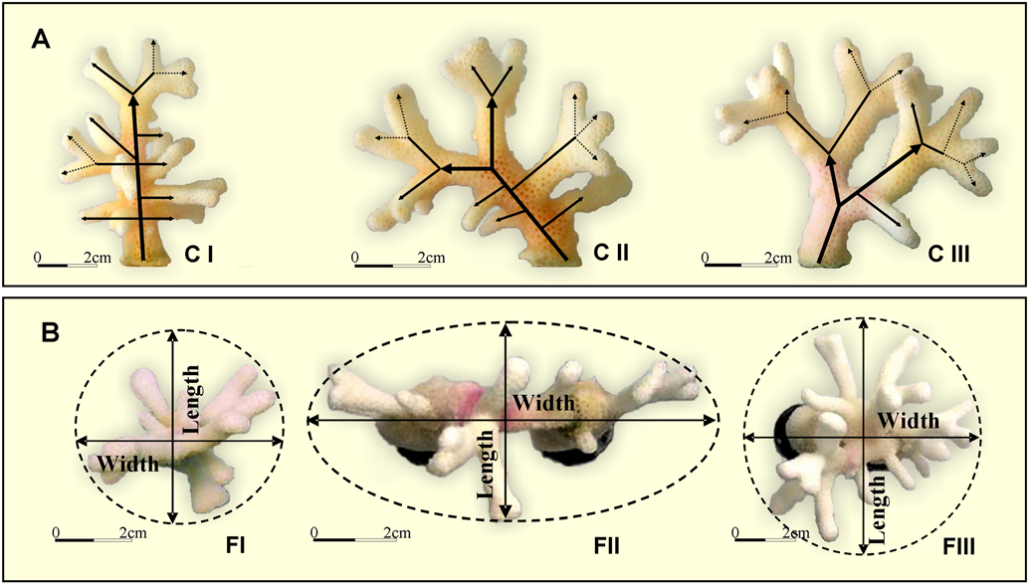
Representative photographs illustrating the typical colonial architectures developed from isolated branches of the seven *Stylophora pistillata* genotypes used in this study (A) Experiment 1: genotype C (CI, a single tip; CII, initial dichotomous tip; CIII, dichotomous tip), depicting the typical colonial architectures developed from branches of genotypes A–D. Bold arrows delineate major growth axes, solid arrows- directionality of N2 branches, dashed arrows- directionality of N3, N4 branches. (B). Experiment 2: genotype F (FI, a single tip; FII, fused double tip; FIII, crossed double tip), depicting the typical colonial architectures developed from branches of genotypes E–G. Dashed circle–the spherical dimension of the colony created by its maximum width and maximum length.

After 1y of *in situ* growth, 88 fragments of experimental set 1 (73.3%) and 48 (66.7%) preparative of experiment 2, developed colonies of different shapes. Each colony was photographed from all sides and all morphometric parameters (Table 1) were calculated. 2-D MDS ordination analysis was performed using two factors: (1) preparative primary shape (experimental group; Fig. 2a, b); (2) origin of genet (source colony; Fig. 2c, d). Preparative in both sets of experiments did not assemble according to the experimental group factor as no significant difference was recorded (ANOSIM: Global R = 0.235, p<0.001 for colonies A, B, C and D; ANOSIM: Global R = 0.184, p = 0.016 for colonies E, F and G; Fig. 2a, b respectively). However, MDS analysis revealed grouping of colonies, which developed in correspondence with the genet factor (ANOSIM: Global R = 0.534, p<0.001 for genets A, B, C and D; ANOSIM: Global R = 0.513, p = 0.016 for genets E, F and G; Fig 2c, d respectively). In the first set of experiments, genet A daughter colonies separated from genets C and D daughter colonies, that clustered into a separate group. Genet B daughter colonies scattered on the MDS plan (Fig. 3c). In the second set of experiments, genet E daughter colonies separated clearly from genet G daughter colonies, while genet F colonies scattered on the MDS plan (Fig. 3d). By analyzing the genet factor in pairwise tests (testing difference between ramets belonging to different genets), we recorded significant differences between genet A to genets B, C and D in experiment 1, and between genets E and G in experiment 2 (Table 2).

**Table 2:** Pairwise test results for dissimilarity of developmental patterns between genets

Donor colony (genet)	N (number of daughter colonies)	R statistics	Significance level (α = 0.05)
A vs. B	18 vs. 23	0.623	0.000
A vs. C	18 vs. 20	0.923	0.000
A vs. D	18 vs. 27	0.826	0.000
B vs. C	23 vs. 20	0.405	0.000
B vs. D	23 vs. 27	0.439	0.000
C vs. D	20 vs. 27	0.146	0.023
E vs. F	13 vs. 14	0.453	0.002
E vs. G	13 vs. 21	0.751	0.000
F vs. G	14 vs. 21	0.313	0.003

Whereas MDS analyses revealed no significant differences between the experimental groups, the general architectural shapes that developed from each of the three different initial morphological shapes in experiment 1 and 2, differ from each other (Fig. 3a, b). In experiment 1, single tip branches developed colonies characterized by a single growth axis structures while the other two groups of bifurcating tips developed colonies that followed two growth axes traits (Fig. 3a). In experiment 2, colonies originating from single tip branches and preparative of crossed double tip formed spherical 3-D architectural structures, while in preparative of fused double-tip, the colonies grew more into 2-D dimension, along the fused tip axis plan (Fig. 3b).

A set of univariate analyses was performed, separately, on each morphometric parameter to further analyze differences in architectures between different treatments within each experiment. In the first set of experiments, the parameters, which were found to be significantly different between the various treatments were: (1) percentage of tip-born branches developed from branch tips (%TBB); (2) percentage of generations 2–4 branches (N2, N3, N4); (3) the ratio of up-growing branches (%UGB) out of all branches. ANOVA results showed significant differences between the complex arrangement groups (p<0.001), except for the percentage of generation 3 branches (N3), in which no significant difference was found between the three settings (Fig. 1a; ANOVA, p>0.05). In the second set of experiments, the parameters were: (1) ratio between maximal widths of colony to its maximal height (Le/H); (2) ratio of up-growing branches (%UGB) out of all branches. ANOVA results revealed significant differences between the three preparative groups (Fig. 1b; ANOVA, p<0.001).

**Figure 4:**
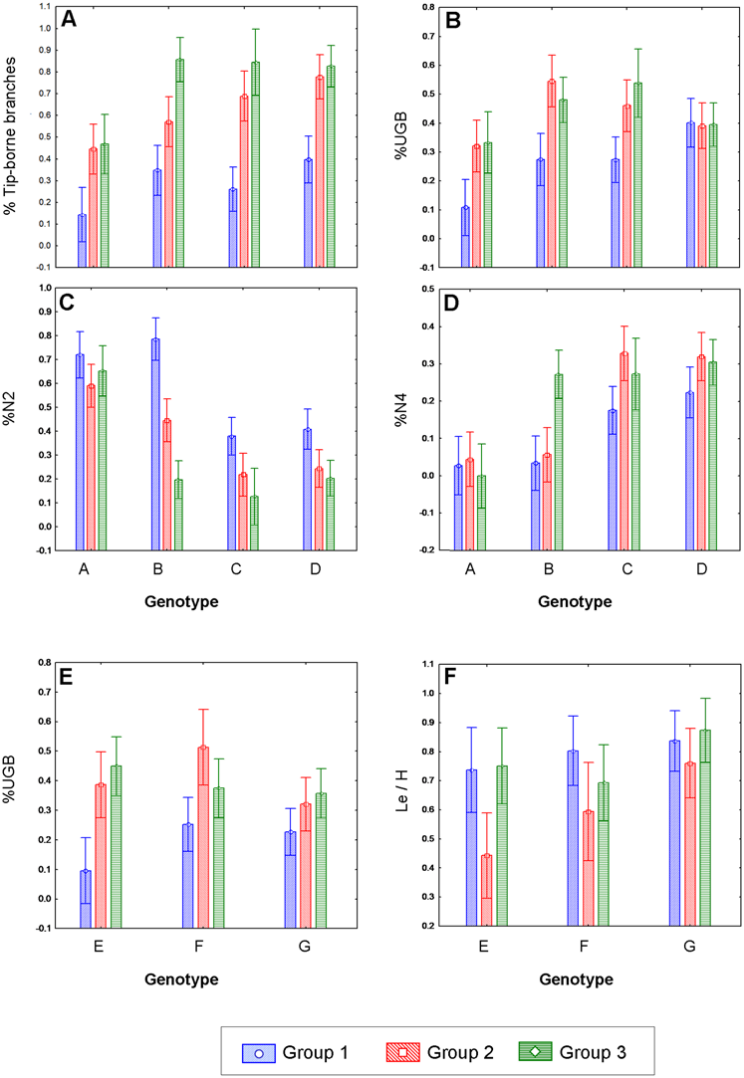
ANOVA results of the morphometric parameters distinguishing between the setting groups (A–D) Experiment 1: group 1 = setting I, single-tip branches; group 2 = setting II, initial dichotomous branches and group 3 = setting III, dichotomous tip branches. (E–F) Experiment 2: group 1 = setting I, single-tip branches; group 2 = preparative II, fused double tips and group 3 = preparative III, crossed double tips. (A) Tip-born branches (TBB). (B) Up-growing branches (UGB). (C) Branches from generation 2 (N2). (D) Branches from generation 4 (N4). (E) Up-growing branches (UGB). (F) Width to height ratio (Le/H).

Univariate analyses for experimental settings (first set of experiments) further revealed that percentages of tip-born branches (%TBB) were lower in setting I (single tip) compared to settings II (initial dichotomous tip) and III (dichotomous tip), an outcome expressed in ramets of all four studied genets (ANOVA; p<0.05, Fig. 4a). This result was repeated in morphometric parameter %UGB (percentages of up-growing branches; ANOVA; p<0.05, Fig. 4b) for colonies developed from genotypes A, B and C, but not for the colonies developed from genotype D (Fig. 4b). Analysis of %N2 and %N4 (percentages of new branches from order 2 and order 4, Fig. 4c, d) revealed no significant differences (p>0.05) between the three setting groups of colony A. However, in colonies developed from genotypes B, C and D, %N2 was higher in setting I compared to settings II and III (p<0.05, Fig 4c). %N4 outcomes varied between the four genotypes; in genotypes C and D %N4 was lower in setting I compared to setting II, but not to setting III that did not differ from either settings (Fig. 4d). In genotype B ramets, setting III outcomes were significantly higher than settings I and II (p<0.05, Fig 4d). Post hoc test (Fisher LSD test) results are summarized in Table 3.

Univariate analyses for experiment 2 preparative, revealed that the ratio between maximal width and maximal length of colony (Le/H) was significantly lower in preparative II in genotypes E and F (p<0.05, Fig. 4e). In ramets developed from genotype G, there were no differences in this parameter between the three preparative (p>0.05, Fig. 4e). The %UGB values (percentages of up-growing branches) were significantly lower in preparative I, compared to preparative II, in genotypes E and F (p<0.05, Fig. 4f), whereas in genotype E similar differences were recorded between preparative I and III (Fig. 4f). Post hoc test (Fisher LSD test) results are summarized in Table 4.

**Table 3:** Post hoc results (Fisher LSD test) evaluating the differences between the experiments settings for each source genotype. sd = significant difference (p<0.05), ns = non significant difference (p>0.05).

Genotype	Pairwise analyses of morphometric parameter in exp. group											
	%TBB			%UGB			%N2			%N4		
	I vs. II	I vs. III	II vs. III	I vs. II	I vs. III	II vs. III	I vs. II	I vs. III	II vs. III	I vs. II	I vs. III	II vs. III
A	sd	sd	ns	sd	sd	sd	ns	ns	ns	ns	ns	ns
B	sd	sd	sd	sd	sd	ns	sd	ns	sd	ns	sd	sd
C	sd	sd	ns	sd	sd	ns	sd	sd	ns	sd	ns	ns
D	sd	sd	ns	ns	ns	ns	sd	sd	ns	sd	ns	ns

**Table 4:** Post hoc results (Fisher LSD test) for the differences between the three experimental settings in each source genotype; sd = a significant difference (p<0.05); ns = non significant difference (p>0.05).

Genotype	Pairwise analyses of morphometric parameter in exp. group					
	%UGB			Le/H		
	I vs. II	I vs. III	II vs. III	I vs. II	I vs. III	II vs. III
E	sd	sd	ns	sd	ns	sd
F	sd	ns	ns	sd	ns	ns
G	ns	sd	ns	ns	ns	ns

## Discussion

**Figure 5:**
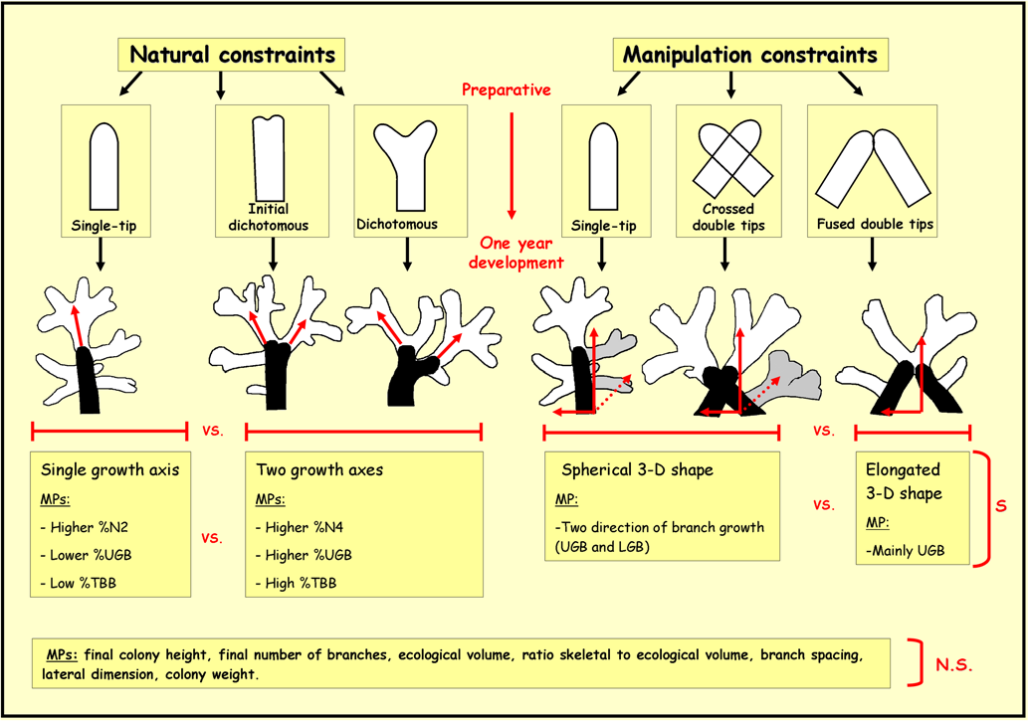
Phenotypic plasticity for the tested morphometric parameters (at the branch and the colonial levels) illustrated by one-year *ex situ* growth of different preparative shapes MP-Morphological parameter; S–Parameters found significantly different between preparatives (p<0.05); N.S.-Parameters found non-significantly different between preparatives (p>0.05).

Morphological characteristics of branching coral forms can be deduced, in general, from characters three hierarchical levels of organization, the individual polyps, the individual branches, and the whole colony entity. Here, *Stylophora pistillata's* colonial structures were approached by evaluating discrete morphometric categories at branch and whole colony levels of organization. Direct comparison of colonial architectures between 136 one-year old colonies (made of seven genotypes) that developed from different branch types and various structural manipulations, revealed plastic morphometric characters at the branch level and predetermined morphometric traits at the colony level (Fig. 5). Out of a suit of 16 morphological parameters tested (10 describing the colony level architecture; 6 at the branch level traits), only four parameters revealed plastic traits of which three were attributed to the branch architectural level (50% of parameters analyzed; up growing branches, tip-born branches, branches from different branch order). The single plastic morphometric parameter on colony level (colony width to height ratio) was revealed after applying extreme, unnatural morphological manipulation, when pairs of isogeneic branches were arranged in tip-to-tip contact. All other nine morphological parameters on colony level (like colony ecological volume, total number of branches, final height etc., Table 1) were kept fixed (major outcomes are shown in Fig. 5). This uncoupling traits expressions, at the two examined hierarchical levels of coral colony organization, allow the developing colony to “play” with plastic geometric structures at the branch level (each relating to the initial fragment type) in order to assemble fixed colonial astogenic trajectories (Fig. 5). Modular growth in *S. pistillata* is therefore an outcome of variations in architectural forms. Any isolated branch is not only the building block of the entire colony [36] but may also initiate various architectures via different morphometric scenarios at the colony level, a phenomenon strictly canalized in *S. pistillata*. Working on branch to colony trajectory in *S. pistillata*, we have recently [31] revealed that colonial astogeny is characterized by a continuum of architectural designs marked by several distinct stages, each representing its own characteristic morphometric parameters. Here, we further elucidated astogenic plasticity associated with traits of initial fragment structures by designing two different sets of experiments. In the first, we observed one-year old colonial architectures developed from small branch fragments with single tip, starting and developing bifurcating tips that were pruned from four *S. pistillata* genotypes. The second examined one-year old colonial architectures developed from artificial structures, each revealing a single or two branch tip axes, pruned from three *S. pistillata* genotypes. Through different traits at branch level, the unalike developmental trajectories (confirming [41] outcomes) in both sets of experiments, culminated in the same pre-planned colonial architecture. Single tip branches, for example, developed more side growing single tip branches, where dichotomous branches, taken from the same donor genotype, developed bifurcating system of branches (Fig. 5).

On the other hand, parameters describing traits at colony level, such as total number of branches, final height of colony, its lateral dimension or ecological volume, did not differ significantly between the experimental groups (Fig. 5). The strictly sessile life of *S. pistillata* facilitates tight integration between all colonial modules, accomplished by creation of various developmental axes and polarities. Similar to different developmental polarities represented by all unitary and colonial cnidarians studied to date [42], [43], [44], [45], [46], different branch types from a single *S. pistillata* colony (up-growing branches, lateral-growing branches; [18], [30] ) or different regions along a branch [20], [32], [34], [47] that represent different physiological capabilities, may also reveal unalike end-point architectural properties. This recalls the arborescent networks of gorgonian octocorals [19], [21], [48]. Similar flexibilities were revealed in response to changes in environmental conditions (e.g., [49]). The ‘reaction norms’ (sensu [49]) studied to-date on corals, evaluated phenotype variations and fitness properties across different environments. Here, we elucidate for the first time, that even different initial fragment structures may have morphometric impacts, and may be considered as reaction norms. For example, in ramets characterized by two branches with a single fused tip, initial growth trajectories developed morphologies along a single plane, creating, during the first step of astogeny (sensu [31]), a 2-D fan like colonial structure, without affecting total ecological volume or branch spacing (Fig. 5). In faster growing genotypes, like genotype G, the spherical 3-D colonial architecture had already been achieved during the astogenic time window of 1 y. In extreme cases (such as in our second set of experiments or in the plate-like *S. pistillata* colonies, growing at 50–60 m depth; unpubl.), the species specific prominent shape cannot be achieved and *S. pistillata* colonies develop other 3-D geometric structures.

Above results, reflect the product of developmental canalization and/or other developmental constraints, where different growth patterns lead to a ‘fixed’ 3-D geometric structure of *S. pistillata* colonies through various growth patterns. By developing different proportions of bifurcating, sub-apical and side-growing branches, astogeny in this species emerges as an integrative and plastic process at the branch level, and does not reflect simple or complex module replications along pre-planned architectural process. This corroborates empirical conclusions [25] that modularity impacts on one level do not necessarily impose impacts on other levels of organization, as well as Foster's [4] results revealing in the coral *Montastraea* no impact between the polyp corallites level and the colonial structure. This may further reflect phenotypic reactions to environmental challenges that are coordinated by regulatory genes working at different hierarchical levels [2]. While coral architecture is presumably related to gene expression, little is known how it is achieved. However, a complex involvement of developmental regulatory genes has recently been elucidated in the ontogeny of a sea anemone [45], hydrozoans colony astogeny [50], and for various colonial cnidarians [42], [44], [45].

As changes at the polyp level have not been studied here, it is possible that the final architectures of *S. pistillata* colonies are related to the traits of the polyp module replication. The developing complex level of within-colony integration, even when accepting the concept of uncoupled developmental patterning at all levels of organization (this work; [48]), reveals a most stringent genetic control of *S. pistillata* complete colonial architecture. Branching colonial forms are often subjected to harsh environmental conditions that not only inflict uneven impacts (light and water gradients, interspecific and intraspecific interactions, etc.; [9], [20], [24]) on different colonial parts, but may cause fragmentation of colonies into different sizes and various structural ramets [51], [52], [53], [54], [55]. At the colony level, chemical signals (“coral isomones”; [23], [32]) are part of the network of controlling mechanisms that lead to fixed colonial architectures. Isolated branch modules, with minimal initial structures, respond differently by exhibiting diverse architectures through different development trajectories, all leading again to the species specific fixed colonial shape. Under normal conditions, the *S. pistillata* rigid 3-D architectural system differs from the congener species *Pocillopora damicornis* [22] that display plastic architectural colonial morphs and striking morphological diversity at the colony level. Similar non-plastic architecture phenomenon was also observed in a massive coral species [12]. Above examples could therefore reveal a myriad of architectural approaches taken by different hermatypic corals, where each level of organization is either genetically controlled or may exhibit various levels of phenotypic plasticity; a mechanism by which coral species may cope with external environmental forces. This meets also the expectations [56] that animals with modular architectures may need to be evaluated with criteria modified from those used to interpret complexity in unitary organisms.
